# Comparative Assessment of PPG-Derived HRV Using MAX30102 Sensor and Analog Circuitry with ADS1115 ADC

**DOI:** 10.3390/s26082487

**Published:** 2026-04-17

**Authors:** Jesús E. Miranda-Vega, Rafael I. Ayala-Figueroa, Yanet Villarreal-González, Pedro A. Escarcega-Zepeda

**Affiliations:** 1 Department of Electrical and Electronic Engineering, Tecnológico Nacional de México/IT Mexicali, Mexicali 21376, Mexico; elias.miranda@itmexicali.edu.mx (J.E.M.-V.); rafaelivan@itmexicali.edu.mx (R.I.A.-F.); 2 Department of Metal-Mechanical Engineering, Tecnológico Nacional de México/IT Mexicali, Mexicali 21376, Mexico; 3 Department of Industrial Engineering, Tecnológico Nacional de México/IT Mexicali, Mexicali 21376, Mexico; pedro.escarcega@itmexicali.edu.mx

**Keywords:** heart rate variability (HRV), photoplethysmography (PPG), analog signal conditioning, active filtering, LM358, ADS1115 ADC, MAX30102 optical sensor, inter-beat interval extraction, Bland–Altman agreement, embedded biosensing systems

## Abstract

Heart rate variability (HRV) is a key physiological marker for autonomic nervous system function and cardiovascular health. Photoplethysmography (PPG) is commonly used to derive HRV metrics in wearable and low-cost monitoring systems. This study presents a comparative assessment of basic HRV metrics obtained from a MAX30102 optical sensor and a custom analog circuitry with an ADS1115 analog-to-digital converter (ADC). Both measurement pathways were carefully aligned using analog high-pass and low-pass filters and a consistent digital filtering pipeline, ensuring that the frequency bands relevant to HRV were preserved. PPG signals were recorded simultaneously, and inter-beat intervals were extracted to calculate the Standard Deviation of NN intervals (SDNN), Root Mean Square of Successive Differences (RMSSD), and Percentage of successive NN intervals >50 ms (pNN50) across multiple 30-s windows. Bland–Altman analysis was employed to evaluate agreement between the two methods. Results indicate that the analog circuit with an ADS1115 achieves comparable HRV basic metrics to the MAX30102 sensor, with improved Signal-to-Noise Ratio (SNR) due to high-resolution ADC and low-noise analog amplification. These findings demonstrate that a carefully designed analog acquisition system can reliably reproduce HRV basic parameters from PPG signals, providing an alternative approach for low-cost, flexible biosensing platforms.

## 1. Introduction

Wearable sensors have emerged as essential tools for continuous health monitoring. They enable non-invasive measurement of physiological signals. Commonly monitored health parameters include heart rate (HR) [1], blood oxygen saturation (SpO_2_) [2], sleep stages [3], electrodermal activity (EDA) [4], blood pressure (BP) [5], respiratory rate [6], skin temperature, and physical activity and motion. In study [7], the authors highlight that wearable devices have a wide range of potential clinical applications, from arrhythmia screening in high-risk individuals to the remote management of chronic conditions such as heart failure and peripheral artery disease.

The use of wearables for physiological monitoring has been well received among aircrew. Personnel are routinely exposed to mild-to-moderate hypoxia, and, anecdotally, the use of wearable devices by pilots in general, commercial, and military aviation operations is increasing [8].

PPG is a well-known technique that has been widely studied due to its comfort and suitability for patient health monitoring. Many wearables, like smartwatches, can provide SpO_2_ and HR based on the PPG technique. A device based on PPG is composed of a light-emitting diode (LED) that emits light and a photodetector that detects the emitted light. The device can be divided into transmissive type and reflective type according to the position of the LED and photodetector [9]. However, what attracts researchers to this technique is not only its use for SpO_2_ or HR measurements; it has also been widely investigated for other applications, such as glucose monitoring. This study [10], provides a comprehensive and systematic evaluation of PPG-based non-invasive blood glucose monitoring. They highlight that near-infrared wavelengths (850–940 nm) in reflective mode demonstrate better glucose sensitivity. The authors [11], proposed a multimodal framework based on electrocardiogram (ECG) and PPG signal fusion to establish a universal blood glucose monitoring model.

HRV derived from PPG provides a non-invasive method to assess autonomic nervous system function and cardiovascular regulation. In wearable applications, accurate HRV estimation depends on proper sensor integration and robust signal processing to minimize motion artifacts and ensure reliable beat detection. The findings reported in [12] support the feasibility of PPG-based HRV monitoring, showing good agreement with ECG-based measurements in healthy individuals, which reinforces its potential for practical and ambulatory assessments.

Recent advances in embedded systems enable the development of improved health monitoring solutions based on wearable applications. This study aims to compare the performance of a MAX30102-based digital PPG sensor with an analog acquisition approach using analog circuitry and the ADS1115 ADC.

The main contributions of this work are as follows:

- Proposing a Bland–Altman analysis to assess the agreement between PPG signals acquired using the MAX30102 integrated sensor and the custom ADS1115-based acquisition system.
- Designing and evaluating a custom PPG circuit in which an LED is implemented as a photosensing element, enabling a low-cost and designer-controllable alternative to fully integrated optical modules.
- Providing an experimental comparison framework that highlights signal quality, measurement agreement, and practical implementation considerations for wearable PPG-based monitoring systems.

The rest of the paper is organized as follows. Section 2 details the materials used to conduct the experiments and describes the methodology employed to evaluate the sensors. Section 3 presents the dataset used in this study. Section 4 reports the experimental results, followed by a discussion in Section 5. Finally, conclusions are provided in Section 6.

## 2. Materials and Methods

In this PPG project, we use the ESP32-S3 (Espressif Systems, Shanghai, China), the MAX30102 (Maxim Integrated, San Jose, CA, USA), and the ADS1115 ADC (Texas Instruments, Dallas, TX, USA). The ESP32-S3 is based on a dual-core Xtensa LX7 (32-bit) CPU running at up to 240 MHz, providing sufficient processing capability for real-time PPG acquisition, digital filtering, and lightweight on-device machine learning. The platform also offers native USB-C connectivity, integrated Wi-Fi and Bluetooth LE, multiple ADC channels, and standard peripherals (SPI, I²C, I²S, UART, and PWM), as well as low-power modes and abundant GPIO resources. We leveraged a 921,600 baud rate to ensure high-throughput and reliable data acquisition of PPG signals.

In this study, we employed the MAX30102, which integrates red (660 nm) and infrared (880 nm) LEDs, photodetectors, ambient-light rejection, and an on-chip FIFO buffer. The sensor supports programmable LED drive currents, pulse-width settings, and sample rates from 50 to 3200 samples per second (sps), and it implements an internal oversampling sigma–delta ADC with high effective resolution (up to 18 bits under optimal conditions). For the experiments reported here, the sampling rate was fixed at 200 sps for both the IR and RED channels to provide adequate temporal resolution for heart-rate analysis while preserving a favorable signal-to-noise tradeoff [13]. Data were read via I²C and buffered to prevent FIFO overruns during continuous acquisition.

The ADS1115 is a low-power, high-precision, 16-bit ADC with an integrated programmable gain amplifier (PGA) that enables accurate acquisition of low-amplitude signals. It supports four single-ended or two differential inputs and communicates via an I²C interface, allowing straightforward integration with embedded platforms. The device provides programmable data rates ranging from 8 to 860 sps and selectable full-scale input ranges, making it well-suited for biomedical and low-frequency sensing applications requiring enhanced resolution and stable digital conversion. In this study, a nominal sampling rate of 250 sps was configured; however, in the firmware, the effective sampling frequency was synchronized to the MAX30102 sampling rate to enable a direct and consistent performance comparison between the two acquisition systems.

The following subsections describe each stage of the proposed pipeline in detail. Section 2.1 explains how MAX30102 signals were processed using digital filtering techniques, including IIR smoothing, DC removal via high-pass filtering, and band-pass filtering within the physiological cardiac frequency range. Section 2.2 describes how ADS1115-based signals were conditioned through an analog front-end consisting of a passive high-pass stage and an active feedback stage implemented with an LM358 operational amplifier. These procedures were designed to enhance signal quality and to support reliable peak detection and subsequent quantitative analysis. The remaining subsections cover data transmission to the computer (Section 2.3), performance metrics (Section 2.4), the experimental setup (Section 2.5), and the peak detection method employed in the experiments (Section 2.6).

### 2.1. Signal Processing Pipeline for MAX30102-Based PPG Signals

The digital processing applied to the PPG signal acquired at a sampling frequency of $f_{s}=200$ sps consists of the following stages:

#### 2.1.1. IIR Smoothing (Exponential Filter)

A first-order IIR filter is applied, defined as follows:

(1) y[n]=αx[n]+(1−α)y[n−1]

where:

- x[n] is the input signal;
- y[n] is the filtered output;
- α is the smoothing coefficient.

This filter attenuates high-frequency noise while maintaining low temporal delay.

#### 2.1.2. High-Pass Filter (DC Removal)

To remove the DC component and slow baseline variations, a first-order high-pass filter with cutoff frequency $f_{c}=0.1$ Hz is used; the sampling frequency is $f_{s}=200$ sps.

A typical discrete implementation is:

(2) $$y_{HP}\left[n\right]=\beta \left(y_{HP}\left[n-1\right]+x\left[n\right]-x\left[n-1\right]\right)$$

The coefficient β is computed as follows:

(3) $$\beta =\frac{\tau }{\tau +T_{s}}$$

where

- τ is the filter time constant (seconds);
- $T_{s}$ is the sampling period (seconds).

with:

(4) $$\tau =\frac{1}{2\pi f_{c}},\;T_{s}=\frac{1}{f_{s}}.$$

#### 2.1.3. Band-Pass Filter (0.1–6 Hz)

The PPG signal is then restricted to the physiological cardiac band using a band-pass filter defined as follows:

(5) 0.1Hz≤f≤6Hz

The general transfer function of an IIR band-pass filter can be expressed as follows:

(6) $$H\left(z\right)=\frac{\sum_{k=0}^{M}b_{k}z^{-k}}{1+\sum_{k=1}^{N}a_{k}z^{-k}}$$

where the coefficients $a_{k}$ and $b_{k}$ determine the filter order and selectivity.

This band covers heart rates from approximately 6 bpm up to 360 bpm. Within this range, the signal retains physiologically relevant cardiac information while attenuating slow baseline drift and motion artifacts [14,15].

#### 2.1.4. Summary MAX30102-Based PPG Signals

The digital conditioning of the MAX30102-based PPG signal can be summarized as follows:

- A first-order IIR exponential filter was applied to attenuate high-frequency noise while preserving the temporal morphology of the waveform.
- A first-order high-pass filter with a cutoff frequency of 0.1 Hz was used to suppress the DC component and slow baseline drift.
- A band-pass filter in the 0.1–6 Hz range was then applied to retain the physiological cardiac band relevant for heart-rate analysis.
- Together, these stages improved signal quality and isolated the pulsatile content prior to peak detection and subsequent metric extraction.

### 2.2. Signal Processing Pipeline for ADS1115-Based PPG Signals

#### 2.2.1. Passive High-Pass Stage

Elements and configuration: a series capacitor (2.2 μF) followed by a resistor to ground (560 kΩ). Resulting behaviour: the network blocks very-low-frequency components (baseline drift) and passes physiological pulsatile content. The nominal corner frequency of this stage is approximately 0.13 Hz, so frequencies well above this value are transmitted with little attenuation.

#### 2.2.2. Active Feedback Stage (LM358)

Elements and configuration: an LM358 whose non-inverting input receives the high-pass output; a feedback branch from the amplifier output to the non-inverting node formed by a resistor (100 kΩ) in parallel with a capacitor (220 nF); the inverting input is referenced to ground through a 4.7 kΩ resistor. Because feedback is returned to the non-inverting input, the topology introduces frequency-dependent positive (regenerative) feedback.

Resulting behavior: the feedback network acts as a low-corner feedback path with a nominal transition near 7.2 Hz. Below this frequency, the resistive feedback term dominates, producing stronger positive feedback and a tendency toward offset or hysteresis. Above this frequency, the capacitive path reduces the steady positive feedback, weakening the regenerative effect and allowing the amplifier to respond more linearly to faster pulsatile components.

Together, the passive high-pass (0.13 Hz) and the feedback network (7.2 Hz) create a band in which physiological pulsations (typical PPG band ≈ 0.5–4 Hz) are passed while very-low-frequency drift is suppressed, and ultra-low-frequency components can be subject to regenerative bias by the active stage. This combination emphasizes preservation of the pulsatile band while allowing controlled baseline shaping at lower frequencies.

**Figure 1:**
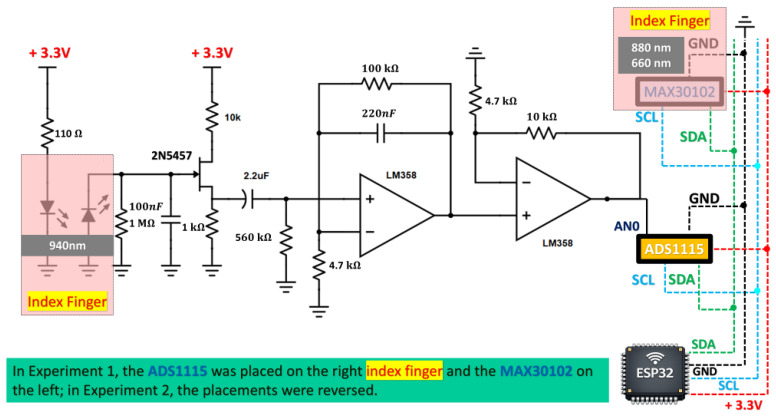
PPG acquisition front-end: 940 nm LED/photodiode illumination and biasing, passive AC coupling (2.2 μF) with 560 kΩ bleed to ground, an LM358 active stage with frequency-dependent feedback (100 kΩ ‖ 220 nF) and 4.7 kΩ reference, followed by a buffering stage and digital back-ends (ADS1115 and MAX30102) interfaced to an ESP32-S3 via I^2^C. Discrete LEDs and components were chosen over fully integrated optical modules to enable customized placement and intensity control, reduce cost, and leverage their widespread commercial availability.

As shown in Figure 1, the proposed PPG acquisition front-end integrates the optical excitation/detection stage with the analog conditioning circuitry. The passive high-pass network and the active LM358 stage, whose filtering characteristics were previously described, are implemented to suppress baseline drift and shape the physiological bandwidth of interest. The active feedback branch (100 kΩ ‖ 220 nF) provides controlled low-frequency conditioning, while the subsequent buffering stage prepares the signal for digitization. Finally, the conditioned output is interfaced to the ADS1115 (940 nm), in parallel with the MAX30102 (880 nm and 660 nm) reference channel, both connected to the ESP32-S3 via I^2^C for synchronized acquisition and processing. It is important to consider that 880 nm has better and deeper penetration in comparison to 940 nm. The present work uses a 940 nm wavelength because it penetrates and is backscattered by deeper vascular structures, such as subcutaneous veins and arteries [16]. Some oximeters that use near-infrared (NIR) LEDs, including wavelengths of 880 nm and 940 nm, exhibit similar ratios of the molar extinction coefficients of Hemoglobin (Hb) and Oxyhemoglobin (${\mathrm{HbO}}_{2}$) [17]. Light with a red wavelength of 640–660 nm and an infrared wavelength of 880–940 nm is mainly used for PPG measurement [18].

#### 2.2.3. Summary ADS1115-Based PPG Signals

The analog conditioning of the ADS1115-based PPG signal can be summarized as follows:

- A passive high-pass stage (2.2μF in series with 560 kΩ to ground) was used to attenuate baseline drift and other very-low-frequency components, with a nominal corner frequency of approximately 0.13 Hz.
- An LM358-based active feedback stage (100 kΩ ‖ 220 nF, with a 4.7 kΩ ground reference) introduced frequency-dependent regenerative feedback and shaped the low-frequency response of the front-end.
- The combined analog stages preserved the physiological pulsatile band of interest while suppressing slow offsets prior to digitization by the ADS1115.

### 2.3. Data Transmission and Visualization in the Graphical User Interface (GUI)

To facilitate real-time monitoring and analysis, the processed data are transmitted to the GUI through a structured binary communication protocol implemented via the BinaryTransport module over a serial interface. In this subsection, the communication architecture is described in detail, including channel mapping, MAX data transmission (Channel 1), ADS data transmission (Channel 2), and the synchronization strategy.

#### 2.3.1. Communication Architecture

All data is streamed through the UART serial interface at a predefined baud rate. Each transmission block follows a structured format composed of four elements: Channel ID, Timestamp, Frame Count, and Payload. The Channel ID specifies the data source (e.g., MAX, ADS, or simulation), the Timestamp provides the time reference obtained from millis(), the Frame Count indicates the number of samples contained in the block, and the Payload consists of binary-encoded sample data. Transmission is carried out using the BinaryTransport::sendBlockBinary routine, which packages and sends the structured data over the serial interface.

This ensures deterministic parsing at the GUI level.

#### 2.3.2. Channel Mapping

Three logical channels are defined:

1. Channel 1: Processed MAX30102 signal (18-bit format, 6 bytes per frame).
2. Channel 2: Processed ADS1115 signal (18-bit equivalent, 3 bytes per sample).

#### 2.3.3. MAX Data Transmission (Channel 1)

After IIR smoothing, DC removal, and band-pass filtering, the MAX signal is organized into frames of six bytes each (representing two 18-bit channels). Each data block is transmitted with channel 1 using the system timestamp, the frame count, and a payload whose size equals six bytes per frame. Detected peak indices, reported with sub-sample precision, are sent separately on channel 4 as 32-bit floating-point values, with a payload length of four bytes per detected peak.

#### 2.3.4. ADS Data Transmission (Channel 2)

The ADS1115 operates in continuous conversion mode at 250 sps. Two consecutive 16-bit samples are averaged to emulate the temporal aggregation of the MAX sensor:

(7) $$x_{\mathrm{avg}}\left[n\right]=\frac{x_{1}\left[n\right]+x_{2}\left[n\right]}{2}$$

The signal is scaled from 16-bit to 18-bit resolution:

(8) $$x_{18}\left[n\right]=\left(x_{\mathrm{avg}}\left[n\right]\ll 2\right)+\mathrm{OFFSET}$$

After identical preprocessing (IIR smoothing, DC removal, and band-pass filtering), a single 18-bit channel is retained and packed into three bytes per sample. Data are transmitted on channel 2 with the system timestamp and ADS frame count, using a payload sized at three bytes per ADS sample. This arrangement guarantees that the GUI receives a number of ADS samples synchronized with the MAX acquisition block.

#### 2.3.5. Synchronization Strategy

The number of ADS samples transmitted per iteration is forced to match the number of MAX frames:

(9) $$f_{\mathrm{ADS}}=f_{\mathrm{MAX}}$$

This guarantees temporal alignment between channels at the GUI level.

#### 2.3.6. Summary GUI

The system implements a deterministic multi-channel binary streaming protocol that ensures:

- Time-aligned acquisition across heterogeneous sensors;
- Fixed-size frame parsing;
- Efficient bandwidth utilization;
- Low-latency real-time visualization in the GUI.

The use of structured binary blocks avoids the overhead associated with text-based protocols and enables precise reconstruction of 18-bit physiological signals at the host side.

### 2.4. Extracted Metrics and Statistical Analysis

Let the detected beat times be $t_{i}$ (s) and the successive inter-beat intervals (IBIs) be:

$${\mathrm{IBI}}_{i}=t_{i+1}-t_{i}\;\left(s\right),\;{\mathrm{IBI}}_{i,\mathrm{ms}}=1000\cdot {\mathrm{IBI}}_{i}\;\left(\mathrm{ms}\right).$$

#### 2.4.1. Time-Domain HRV Metrics

(10) $$\begin{array}{c} \mathrm{SDNN}=\mathrm{std}\left({\mathrm{IBI}}_{\mathrm{ms}}\right)\;\left(\mathrm{ms}\right)\; \end{array}$$

(11) $$\begin{array}{c} \mathrm{RMSSD}=\sqrt{\frac{1}{N-1}\sum_{i=1}^{N-1}{\left({\mathrm{IBI}}_{(i+1),\mathrm{ms}}-{\mathrm{IBI}}_{i,\mathrm{ms}}\right)}^{2}}\;\left(\mathrm{ms}\right) \end{array}$$

(12) $$\begin{array}{c} \mathrm{pNN}50=100\cdot \frac{\#\{i:|{\mathrm{IBI}}_{(i+1),\mathrm{ms}}-{\mathrm{IBI}}_{i,\mathrm{ms}}|>50\}}{N-1}\;(\%)\; \end{array}$$

where *N* is the number of IBI samples.

#### 2.4.2. Beat/Rate Metrics


(13) $$\begin{array}{c} \mathrm{NumPeaks}=\#\{\mathrm{detected}\;\mathrm{beats}\}\; \end{array}$$

(14) $$\begin{array}{c} {\mathrm{BPM}}_{i}=\frac{60}{{\mathrm{IBI}}_{i}}\;(\mathrm{beats}\;\mathrm{per}\;\mathrm{minute})\; \end{array}$$

(15) $$\begin{array}{c} \bar{\mathrm{BPM}}=\mathrm{mean}\left({\mathrm{BPM}}_{i}\right),\;\mathrm{StdBPM}=\mathrm{std}\left({\mathrm{BPM}}_{i}\right) \end{array}$$

#### 2.4.3. Inter-Sensor Agreement and Comparison

Pairing of the beat-by-beat series from two sensors (MAX vs. ADS) was performed by interpolation onto a common time grid prior to comparison.

Intraclass correlation coefficient (ICC) (Shrout & Fleiss ICC(2,1)):

$$\mathrm{ICC}\left(2,1\right)=\frac{\mathrm{MSR}-\mathrm{MSE}}{\mathrm{MSR}+\left(k-1\right)\mathrm{MSE}+\frac{k(\mathrm{MSC}-\mathrm{MSE})}{n}}$$

where MSR (mean square for rows), MSC (mean square for columns), and MSE (mean square error) are ANOVA components for an n×k data matrix.

Bland–Altman analysis: for paired series x,y define the differences $d_{i}=x_{i}-y_{i}$. The mean bias and limits of agreement (LoA) are:

$$\mathrm{bias}=\bar{d},\;\mathrm{LoA}=\mathrm{bias}\pm 1.96\cdot \mathrm{sd}\left(d\right).$$

Beat-to-beat Pearson correlation: the Pearson correlation coefficient is:

$$r=\frac{\sum_{i}\left(x_{i}-\bar{x}\right)\left(y_{i}-\bar{y}\right)}{\sqrt{\sum_{i}{\left(x_{i}-\bar{x}\right)}^{2}}\sqrt{\sum_{i}{\left(y_{i}-\bar{y}\right)}^{2}}},$$

and the two-sided *p*-value can be computed from the *t*-statistic $t=r\sqrt{\left(n-2\right)/\left(1-r^{2}\right)}$ with n−2 degrees of freedom.

##### Notes

- IBIs outside physiological limits (e.g., IBI<0.300 s or IBI>2.000 s) were treated as outliers and corrected via local interpolation prior to metric computation.
- When needed, consecutive ADS samples were averaged and amplitude-scaled to produce an 18-bit equivalent stream prior to identical preprocessing and metric extraction, ensuring comparable effective resolution between acquisition systems

### 2.5. Experimental Setup and Configuration

This section describes the experimental setup and acquisition configuration used for the bilateral comparison of the two PPG systems. It outlines the sensor placement, the two experimental sessions, and the recording conditions employed to ensure a consistent and reproducible evaluation framework.

**Figure 2:**
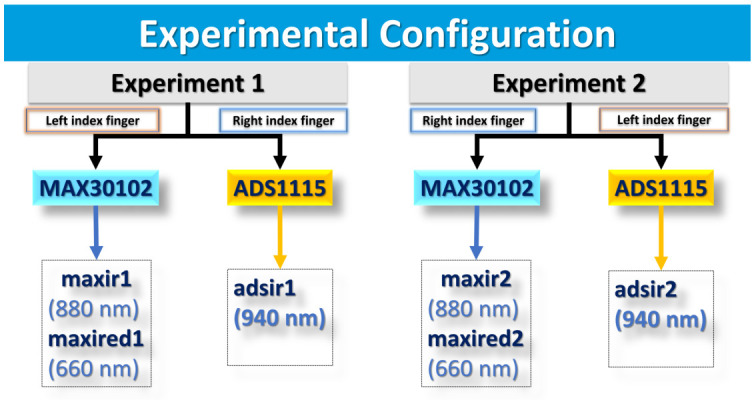
Experimental configuration for the bilateral comparison of the two PPG acquisition systems.

Figure 2 shows the experimental configuration used in this study. Two experiments were conducted in separate sessions. In both experiments, PPG signals were acquired using two channels from the MAX30102 and one channel from the ADS1115. In Experiment 1, the MAX30102 sensor was placed on the left index finger, while the ADS1115-based acquisition system was placed on the right index finger. In Experiment 2, the configuration was reversed, with the MAX30102 placed on the right index finger and the ADS1115 system on the left index finger.

### 2.6. Hybrid Peak Detection Pipeline

There is extensive literature on peak detection methods based on prominence [19], sliding-window recovery [9], and template matching [20]. In this work, a hybrid adaptive peak detection method was used by combining the aforementioned approaches. The procedure is summarized as follows:

- The signal was first filtered and lightly smoothed to reduce noise and remove the DC component.
- An initial peak detection step was performed using the MATLAB R2024b function findpeaks.
- Progressive prominence thresholds were applied to retain reliable peaks.
- A sliding-window search was used to recover weak or missed peaks.
- A morphological beat template was constructed from the detected beats.
- Large gaps in the IBIs were inspected to identify missed beats.
- Candidate peaks were validated using the first derivative, peak prominence, and template correlation.
- Duplicate detections were removed using a refractory period.
- Possible double-beat detections were corrected.
- The final IBIs intervals were cleaned before HR and HRV metrics were computed.

### 2.7. SNR Analysis

In this study, the SNR was computed for each signal using five non-overlapping 60 s segments. Both Experiment 1 and Experiment 2 had a total duration of 5 min per subject. Before processing, non-finite values were removed, and linear detrending was applied. A fourth-order Butterworth bandpass filter (0.5–5.0 Hz) was then used to isolate the cardiac frequency band. The power spectral density (PSD) was estimated using Welch’s method. The dominant frequency was identified as the main peak within the cardiac band. Signal power was defined by integrating the PSD within a narrow band of ±0.15 Hz around this peak. Noise power was computed by integrating the remaining portion of the cardiac band, excluding the signal band. Finally, the SNR was calculated as follows:

(16) $$\mathrm{SNR}=10\log_{10}\left(\frac{P_{\mathrm{signal}}}{P_{\mathrm{noise}}}\right).$$

Results were summarized as mean ± standard deviation for each signal and experiment.

## 3. Dataset Description

The dataset comprises synchronized PPG recordings acquired simultaneously from two channels: MAX30102 provides dual-wavelength measurements (RED = 660 nm; IR = 880 nm), whereas the ADS1115, using an ADS analog front end, provides infrared measurements only. Data were stored in comma-separated values (CSV) format. The acquisition system supports both paired samples (RED/IR from the MAX30102) and single-value IR samples (IR from the ADS1115), ensuring compatibility across heterogeneous hardware configurations.

**Figure 3:**
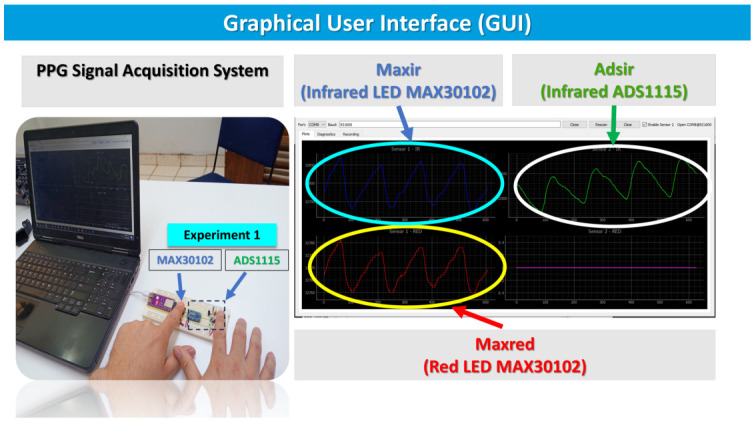
PPG signal acquisition at rest using both index fingers (Experiment 1). The GUI displays three channels: IR (880 nm) and RED (660 nm) signals from the MAX30102 sensor, and an IR (940 nm) channel acquired via the ADS1115. The PPG signal from the MAX30102 appears inverted relative to the ADS1115 signal; the systolic peak corresponds to the highest point of the PPG waveform [21,22].

Figure 3 illustrates the PPG signal acquisition process during a resting condition using both index fingers placed on two optical sensing modules connected to a computer-based GUI. The left panel shows the experimental setup, where the subject places the index fingers on the sensors mounted on a prototyping board while the signals are visualized in real time on a laptop. It is important to note that each index finger produces a different PPG signal, which is captured by its respective ADC (MAX30102 and ADS1115). The GUI displays three active acquisition channels: two channels corresponding to the MAX30102 optical sensor, which provide the infrared (IR) and red LED PPG signals, and an additional single channel obtained from an ADS1115 analog-to-digital converter, configured for 940 nm photoplethysmographic acquisition. In the interface, the IR signal from the MAX30102 is highlighted in the upper-left plot, the red PPG signal from the same sensor appears in the lower-left plot, and the ADS1115-based 940 nm channel is displayed in the upper-right plot. These channels allow simultaneous visualization and comparison of multiple PPG signals acquired under resting physiological conditions.

Buffered visualization employed circular queues, and file-writing operations were protected by a thread-lock mechanism to prevent race conditions, thereby guaranteeing data integrity during real-time acquisition. To ensure reproducibility, the dataset documentation reports sensor specifications, acquisition settings (sampling frequency and ADC resolution), recording duration, the number of sessions and subjects, and experimental conditions, including sensor placement and motion constraints. Preprocessing steps, when applied, are explicitly described and include filtering, baseline correction, and motion-artifact mitigation.

## 4. Results

### 4.1. HRV and HR Metrics with Photosensors MAX30102 and ADS1115

This section presents the HRV and HR metrics obtained from five subjects across two experimental segments. The analysis focuses on the consistency of measurements between the MAX-based photosensors and the ADS acquisition system, as well as the influence of experimental conditions on signal stability and peak detection performance.

As shown in Table 1, for Subject 1, the HRV and HR metrics demonstrate strong consistency across both the MAX-based PPG experimental configurations (maxir1, maxred1, maxir2, and maxred2) and the ADS acquisition experimental configurations (ads1 and ads2). Note that ads1 corresponds to Experiment 1, and ads2 corresponds to Experiment 2. Each experiment uses only one channel (940 nm) with the ADS1115. Maxir1 and maxred1 correspond to Experiment 1, and maxir2 and maxred2 correspond to Experiment 2, using the MAX30102 sensor.

Mean HR remains remarkably stable around 87.2–87.6 BPM for all signals, with comparable standard deviations (≈3.0–3.7 BPM), indicating similar beat-to-beat variability capture across hardware platforms. Time-domain HRV indices (SDNN ≈ 23–30 ms; RMSSD ≈ 14.7–16.9 ms) also exhibit close agreement between MAX and ADS measurements within each segment, suggesting coherent IBI interval detection performance. Minor differences in pNN50 values likely reflect threshold sensitivity rather than systematic sensor bias. Overall, the results support measurement consistency and comparable physiological trend representation between the MAX sensor modules and the ADS-based acquisition system for this subject.

Table 2 shows the results for Subject 2, Experiment 1, demonstrating strong inter-sensor consistency, with closely aligned SDNN (42.5–42.8 ms), RMSSD (31–35 ms), mean HR (93.4–93.5 BPM), and comparable HR dispersion (6.17–6.20 BPM). This agreement suggests stable IBI interval detection and coherent physiological representation across both MAX-based PPG channels and the ADS acquisition system under controlled conditions. In contrast, Experiment 2 exhibits substantially larger variability in SDNN and RMSSD values, along with differences in peak counts and dispersion metrics. These discrepancies may reflect altered measurement dynamics, potentially associated with sustained or uneven index finger pressure on the sensor, which can influence peripheral perfusion and waveform morphology, thereby affecting beat-to-beat interval estimation.

Table 3 presents the HRV and HR metrics for Subject 3. It is important to emphasize that both experimental segments (Experiment 1 and Experiment 2) demonstrate strong inter-sensor and intra-experimental consistency. In Experiment 1, SDNN (81.5–81.8 ms), RMSSD (63.6–64.1 ms), and mean HR (73.2 BPM) are nearly identical across the MAX (IR and Red) and ADS channels, indicating stable IBI interval detection and coherent physiological representation. Similarly, Experiment 2 preserves close agreement among sensors, with SDNN (81.0–81.4 ms), RMSSD (46.8–48.0 ms), and mean HR (75.7 BPM) remaining highly aligned. These findings suggest that Subject 3 may have maintained constant pressure on the sensors and remained focused during the experiment, thereby minimizing involuntary movements.

Table 4 shows strong consistency between the MAX (IR and Red) channels and the ADS acquisition system in both experiments. SDNN, RMSSD, and mean BPM values are closely aligned across sensors within each segment. This agreement indicates stable IBI interval estimation and coherent HRV parameter extraction. The similarity of peak counts further supports reliable beat detection. Such consistency suggests that the subject likely maintained adequate finger pressure and concentration during the recordings. Stable acquisition conditions facilitate the performance and robustness of peak detection algorithms.

Table 5 presents the results for Subject 5 and reveals a clear divergence between the two experimental segments. In Experiment 1, strong agreement is observed among the MAX (IR and Red) channels and the ADS system, with closely aligned SDNN, RMSSD, mean BPM, and peak counts. This indicates stable acquisition conditions and consistent IBI interval estimation across sensors. In contrast, Experiment 2 exhibits pronounced discrepancies, particularly between the MAX channels and the ADS system. The markedly elevated SDNN, RMSSD, and pNN50 values in the MAX signals, together with increased HR dispersion, suggest waveform distortion or instability. These findings are consistent with the presence of excessive or uneven finger pressure during the crossover phase of the second experiment, which may have altered peripheral perfusion and affected peak detection reliability. Overall, the results highlight the sensitivity of HRV metrics to sensor contact conditions and emphasize the importance of controlled pressure for robust signal acquisition and algorithmic performance.

### 4.2. Time-Domain PPG Signals Measured with Max and ADS Photosensors

**Figure 4:**
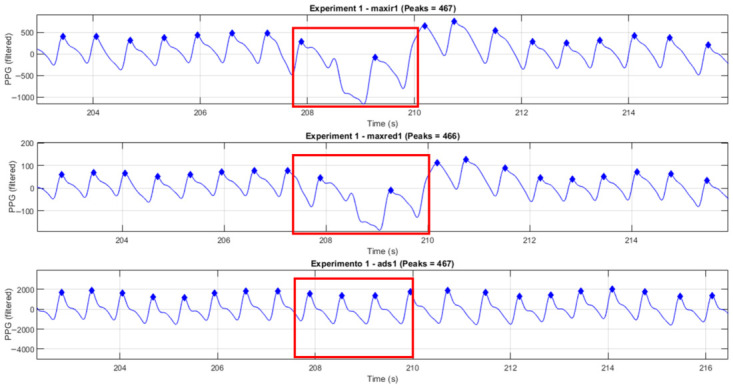
PPG signals from Experiment 1 (Subject 2) for maxir1, maxred1, and ads1. The red box highlights a segment affected by motion artifact or pressure disturbance, showing waveform distortion and peak alteration.

**Figure 5:**
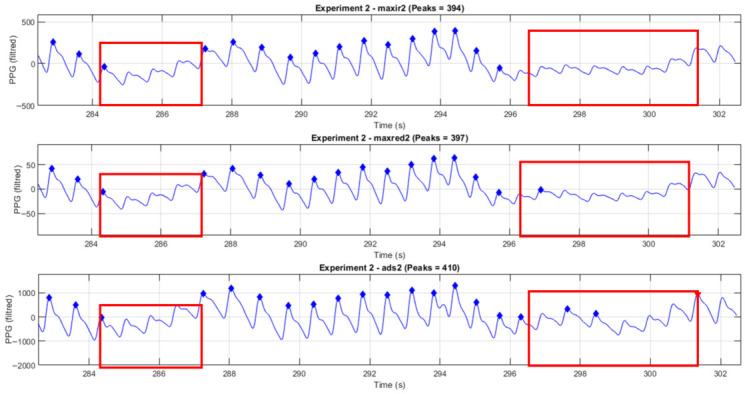
PPG signals from Experiment 2 (Subject 2) for maxir1, maxred1, and ads1. The red box highlights a segment affected by motion artifact or pressure disturbance, showing waveform distortion and peak alteration.

The following Figure 4 and Figure 5 show, in the time domain, the segments where motion artifacts interfered with the measurements.

Figure 4 depicts the waveforms from Subject 2. The waveform distortion is clearly observable. The red rectangle highlights a segment affected by motion artifacts or improper pressure, where noticeable waveform deformation and peak irregularities occur. This highlighted region also reveals variations in signal amplitude and morphology that may affect peak detection accuracy and subsequent HRV analysis. In Experiment 1, the ads1 signal demonstrated better performance than maxir1 and maxred1, detecting one additional peak. Although Experiment 1 was not considerably affected by this disturbance, the results for the same subject in Experiment 2 (see the following figure) were not satisfactory. It is important to mention that this study is not focused on peak detection; we are interested in the performance of the ADS1115 compared to the MAX30102 sensor.

Figure 5 depicts the waveforms from Subject 2. Many peaks are not detected accurately. Upon reviewing the entire waveform, the algorithms in this study correctly detected only 394, 397, and 410 peaks for maxir2, maxred2, and ads2, respectively.

### 4.3. Bland–Altman Analysis of BPMs Derived from MAX30102 and ADS1115 Measurements

**Figure 6:**
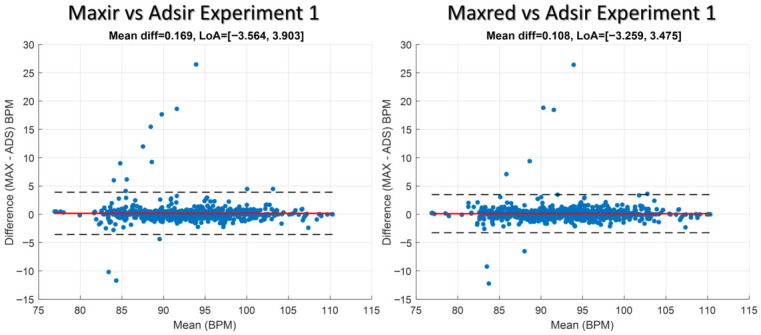
Bland–Altman plot for Experiment 1 comparing BPM estimates derived from the MAX (maxir1 and maxred1) and ADS1115 (ads1) sensors.

**Figure 7:**
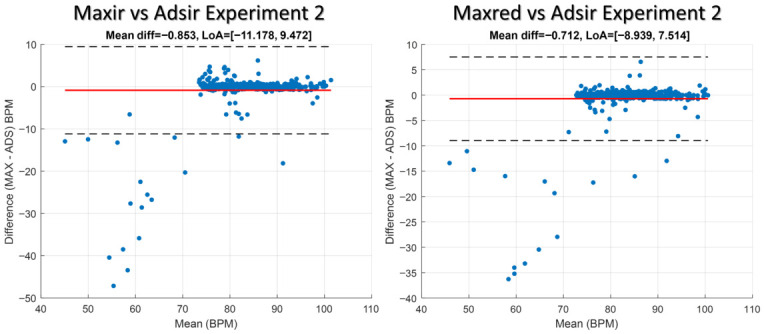
Bland–Altman plot for Experiment 2 comparing BPM estimates derived from the MAX (maxir2 and maxred2) and ADS1115 (ads2) sensors.

Figure 6 and Figure 7 show Bland–Altman plots for Experiments 1 and 2, respectively. The x-axis represents the mean BPM of the paired measurements, while the y-axis indicates the difference (MAX–ADS) in BPM. The red line denotes the mean bias, and the dashed black lines represent the 95% limits of agreement. Points lying outside these limits correspond to outliers, highlighting measurements where the two sensors differ substantially. Experiment 1 shows the analysis that was performed by comparing the MAX signals (maxir1 and maxred1) with the ADS1115 signal (ads1). Experiment 2 shows the analysis that was performed by comparing the MAX signals (maxir2 and maxred2) with the ADS1115 signal (ads2). A post-processing algorithm must be applied to mitigate outliers by utilizing a signal quality index derived from metrics such as SNR, perfusion index, waveform skewness and kurtosis, pulse amplitude variability, baseline wander, and spectral features, among others.

### 4.4. ICC Comparison by Subject Between MAX30102 and ADS1115

To evaluate the reliability of the measurements across subjects and experimental conditions, ICCs were computed between signals acquired with MAX30102 (IR/RED) and ADS1115 (IR). This analysis provides a subject-level perspective on agreement, highlighting both the overall consistency of the acquisition methods and the variability that may arise from individual or contextual factors.

**Figure 8:**
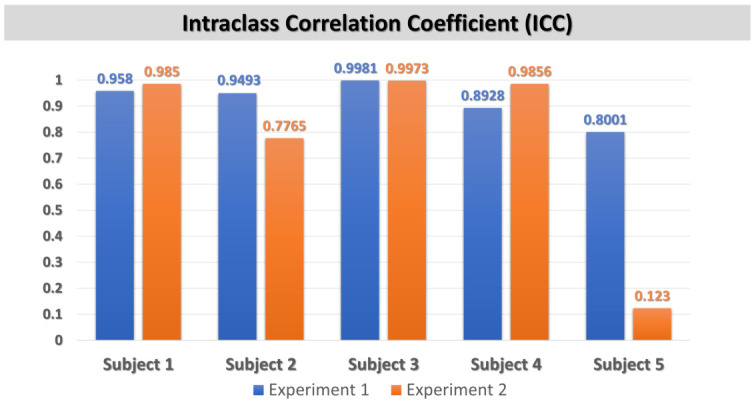
ICC values per subject for two experimental conditions, comparing measurements obtained from the MAX30102 (IR/RED) and the ADS1115 (IR) acquisition.

Figure 8 illustrates the ICC analysis and demonstrates strong overall agreement between the MAX30102 (IR/RED) and ADS1115 (IR) measurements across subjects, with most values exceeding 0.89, indicating excellent reliability. Notably, Subjects 1, 3, and 4 exhibit consistently high agreement, with ICC values greater than 0.95 in at least one experiment. However, substantial variability is observed in Subject 2 (drop to 0.7765) and especially Subject 5 (ICC = 0.123 in Experiment 2), suggesting sensitivity to subject-specific factors, signal quality, or experimental conditions.

### 4.5. Bland–Altman Analysis of HRV Metrics Derived from MAX30102 and ADS1115 Measurements

We have performed a Bland–Altman analysis for the HRV metrics (SDNN, RMSSD, and pNN50) to evaluate the agreement between the MAX30102 and ADS1115 acquisition systems (see Table 6 and Table 7). The analysis was conducted separately for each phase and signal (MAX IR and MAX RED vs. ADS), using 5 min segments per subject. Results showed a high level of agreement during Experiment 1, with negligible bias and narrow limits of agreement across all HRV metrics. In Experiment 2, larger dispersion was observed, which is attributed to physiological and optical variability introduced by the change in finger location rather than differences in acquisition hardware performance. High agreement was observed in Experiment 1 according to the Bland–Altman analysis, while the increased variability in Experiment 2 is attributed to finger repositioning rather than to hardware differences.

### 4.6. Quantitative SNR Analysis of MAX30102 and ADS1115 Measurements

This subsection presents a quantitative analysis of the SNR to compare the performance of the MAX30102 and ADS1115-based PPG acquisition systems across experimental conditions.

**Figure 9:**
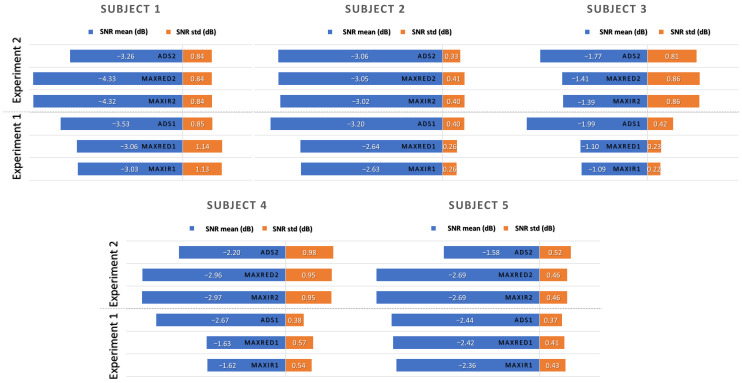
Comparison of the mean and standard deviation of the SNR for MAX30102 (IR and RED) and ADS1115-based measurements across Experiments 1 and 2.

Figure 9 shows the comparison of the mean and standard deviation of the SNR for MAX30102 (IR and RED) and ADS1115-based measurements across Experiments 1 and 2. Overall, the ADS1115-based acquisition system demonstrated competitive SNR performance across subjects and experiments. Although the MAX channels occasionally achieved slightly higher mean SNR in Experiment 1, the ADS signals often exhibited lower standard deviation, indicating more stable behavior. In Experiment 2, ADS matched or surpassed MAX in mean SNR for several subjects—particularly Subjects 1, 4, and 5—while remaining comparable in variability. These results suggest that the proposed ADS-based approach can provide robust and consistent PPG acquisition, despite its lower native resolution.

## 5. Discussion

The results demonstrate that, under stable acquisition conditions, the MAX30102 modules and the ADS1115-based acquisition system produce highly consistent HR and time-domain HRV metrics, while deviations arise when signal quality is compromised. For example, we can observe that in Subjects 1, 3, and 4, mean BPM, SDNN, RMSSD, and peak counts were closely aligned across MAX and ADS channels, indicating comparable IBI-interval detection and robust beat-detection performance when finger contact and motion were controlled. By contrast, Subjects 2 and 5 exhibited pronounced discrepancies in the second experimental segment, characterized by elevated SDNN/RMSSD and altered peak counts in the MAX signals, which we attribute to waveform distortion caused by uneven or excessive finger pressure and motion artifacts.

The Bland–Altman analysis corroborates these observations: most paired measurements cluster near the mean bias, but outliers beyond the 95% limits of agreement identify specific recordings in which sensor disagreement is substantial. These outliers coincide with visually identified waveform distortions (Subject 2) and with sessions showing large increases in SDNN and RMSSD (Subject 5), supporting the interpretation that contact pressure and motion are primary drivers of inter-sensor divergence rather than intrinsic systematic bias between the hardware platforms.

In the case of the SNR analysis, the ADS1115 was robust in Experiment 2 with Subject 5, which presented inconsistent HRV values and abnormal measurements, according to the results shown in Table 5. In this experiment, the MAX30102 measurements for this subject appear susceptible to motion artifacts, which may have influenced the results.

The limitations of this study can be summarized. First, the dataset included a relatively small number of subjects, which reduces statistical power and may limit the generalizability of the findings. Second, the short duration of the PPG recordings limits the clinical relevance of the HRV metrics. Third, the recordings were of short duration, restricting the assessment of signal stability and temporal variability. Fourth, a controlled evaluation of finger contact pressure was not performed; given that contact pressure can significantly affect PPG signal quality, its absence represents an important uncontrolled source of variability. Fifth, a post-processing signal quality index (SQI) and robust peak-detection methods should be employed to enhance overall performance.

## 6. Conclusions

This study contributes a compact yet practical framework for evaluating wearable PPG acquisition systems. The proposed PPG circuit, which uses an LED as a photosensing element, demonstrates a low-cost and designer-configurable alternative to fully integrated optical modules. Overall, the experimental comparison highlights key aspects of signal quality, measurement agreement, and implementation feasibility for wearable PPG-based monitoring applications. The ADS1115 and MAX30102 systems yield comparable HR and HRV metrics under controlled conditions, but their agreement degrades predictably with motion and contact disturbances; addressing these practical sources of variability is essential for reliable cross-platform PPG monitoring.

The ICC results demonstrate strong reliability between the MAX30102 and ADS1115 measurements, with most values exceeding the threshold for excellent agreement. While high consistency was observed across several subjects, variability in others underscores the influence of subject-specific factors and experimental conditions. These findings highlight both the robustness of the approach and the need to account for individual differences when assessing measurement reliability.

The ADS1115 showed notable robustness despite its 16-bit native resolution. Upsampling narrowed the effective bit-depth gap with the MAX30102. Software preprocessing further compensated for lower hardware resolution. Performance matched the MAX sensor in stable segments. In agreement with the SNR analysis, the ADS1115-based system exhibited competitive performance in several experiments, and in some cases even outperformed the MAX30102. These results suggest that the proposed acquisition approach can provide reliable PPG measurements despite its lower native hardware resolution.

Future work will focus on using ECG as the gold standard for HRV analysis while simultaneously employing these sensors in experimental studies to improve the circuit design and develop a robust, Bluetooth-enabled wearable device; to this end, we plan to record synchronous ECG and PPG signals, enabling beat-to-beat alignment and direct comparison of HR and HRV metrics.

## Figures and Tables

**Table 1:** Subject 1—HRV and PPG metrics.

**Experiment 1**						
**Signal**	**SDNN (ms)**	**RMSSD (ms)**	**pNN50 (%)**	**NumPeaks**	**Mean BPM**	**Std BPM**
maxir1	23.67	16.73	0.91	437	87.52	3.05
maxred1	23.74	16.95	0.68	437	87.49	3.06
ads1	24.28	15.90	0.00	440	87.58	3.14
**Experiment 2**						
**Signal**	**SDNN (ms)**	**RMSSD (ms)**	**pNN50 (%)**	**NumPeaks**	**Mean BPM**	**Std BPM**
maxir2	30.07	15.19	0.23	434	87.19	3.74
maxred2	29.73	14.75	0.23	434	87.21	3.70
ads2	29.99	15.29	0.23	435	87.23	3.74

**Table 2:** Subject 2—HRV and PPG metrics.

**Experiment 1**						
**Signal**	**SDNN (ms)**	**RMSSD (ms)**	**pNN50 (%)**	**NumPeaks**	**Mean BPM**	**Std BPM**
maxir1	42.52	34.78	12.90	467	93.53	6.16
maxred1	42.82	34.39	12.28	466	93.50	6.19
ads1	42.72	31.39	9.24	467	93.36	6.17
**Experiment 2**						
**Signal**	**SDNN (ms)**	**RMSSD (ms)**	**pNN50 (%)**	**NumPeaks**	**Mean BPM**	**Std BPM**
maxir2	104.50	97.04	16.58	394	84.37	7.96
maxred2	84.44	77.40	18.73	397	84.46	7.44
ads2	59.51	46.59	18.38	410	84.62	6.72

**Table 3:** Subject 3—HRV and PPG metrics.

**Experiment 1**						
**Signal**	**SDNN (ms)**	**RMSSD (ms)**	**pNN50 (%)**	**NumPeaks**	**Mean BPM**	**Std BPM**
maxir1	81.68	63.74	42.14	365	73.23	7.41
maxred1	81.49	63.58	41.59	365	73.22	7.39
ads1	81.84	64.12	40.93	366	73.25	7.41
**Experiment 2**						
**Signal**	**SDNN (ms)**	**RMSSD (ms)**	**pNN50 (%)**	**NumPeaks**	**Mean BPM**	**Std BPM**
maxir2	81.06	47.08	29.25	378	75.76	7.69
maxred2	81.04	46.83	28.19	378	75.76	7.69
ads2	81.38	47.99	30.13	377	75.74	7.71

**Table 4:** Subject 4—HRV and PPG metrics.

**Experiment 1**						
**Signal**	**SDNN (ms)**	**RMSSD (ms)**	**pNN50 (%)**	**NumPeaks**	**Mean BPM**	**Std BPM**
maxir1	39.41	33.89	12.11	423	84.33	4.66
maxred1	39.72	35.74	13.22	418	84.36	4.72
ads1	39.64	28.28	5.92	424	84.41	4.74
**Experiment 2**						
**Signal**	**SDNN (ms)**	**RMSSD (ms)**	**pNN50 (%)**	**NumPeaks**	**Mean BPM**	**Std BPM**
maxir2	34.11	21.94	1.84	435	86.47	4.42
maxred2	34.03	21.92	1.84	435	86.48	4.42
ads2	34.07	22.33	1.38	435	86.57	4.45

**Table 5:** Subject 5—HRV and PPG metrics.

**Experiment 1**						
**Signal**	**SDNN (ms)**	**RMSSD (ms)**	**pNN50 (%)**	**NumPeaks**	**Mean BPM**	**Std BPM**
maxir1	35.78	36.51	13.74	344	68.28	2.85
maxred1	35.77	36.56	12.57	344	68.28	2.86
ads1	34.78	35.53	9.94	344	68.27	2.80
**Experiment 2**						
**Signal**	**SDNN (ms)**	**RMSSD (ms)**	**pNN50 (%)**	**NumPeaks**	**Mean BPM**	**Std BPM**
maxir2	112.70	178.60	76.20	313	63.34	7.55
maxred2	110.40	180.30	76.12	312	63.58	7.54
ads2	43.84	51.06	33.12	316	62.60	2.89

**Table 6:** Bland–Altman analysis of HRV metrics in Experiment 1.

HRV Metric	MAX_IR vs. ADS: Bias [LoA]	MAX_RED vs. ADS: Bias [LoA]
SDNN	−0.04 ms [−1.24, 1.15]	0.05 ms [−1.11, 1.22]
RMSSD	2.08 ms [−2.62, 6.79]	2.40 ms [−3.67, 8.47]
pNN50	3.16% [−1.07, 7.39]	2.86% [−2.44, 8.17]

**Table 7:** Bland–Altman analysis of HRV metrics in Experiment 2.

HRV Metric	MAX_IR vs. ADS: Bias [LoA]	MAX_RED vs. ADS: Bias [LoA]
SDNN	22.73 ms [−40.65, 86.11]	18.17 ms [−38.98, 75.32]
RMSSD	35.32 ms [−74.59, 145.22]	31.59 ms [−78.70, 141.88]
pNN50	8.17% [−30.12, 46.46]	8.38% [−29.62, 46.37]

## Data Availability

The data presented in this study are available upon request from the corresponding author.
